# Supplementary material for: High-throughput RNA structure probing reveals critical folding events during early 60S ribosome assembly in yeast
Source: Nat Commun. 2017 Sep 28;8:714. doi: 10.1038/s41467-017-00761-8 (PMC5620067; doi:10.1038/s41467-017-00761-8)
Supplement: Supplementary file 1 — Supplementary Information [file 41467_2017_761_MOESM1_ESM.pdf]

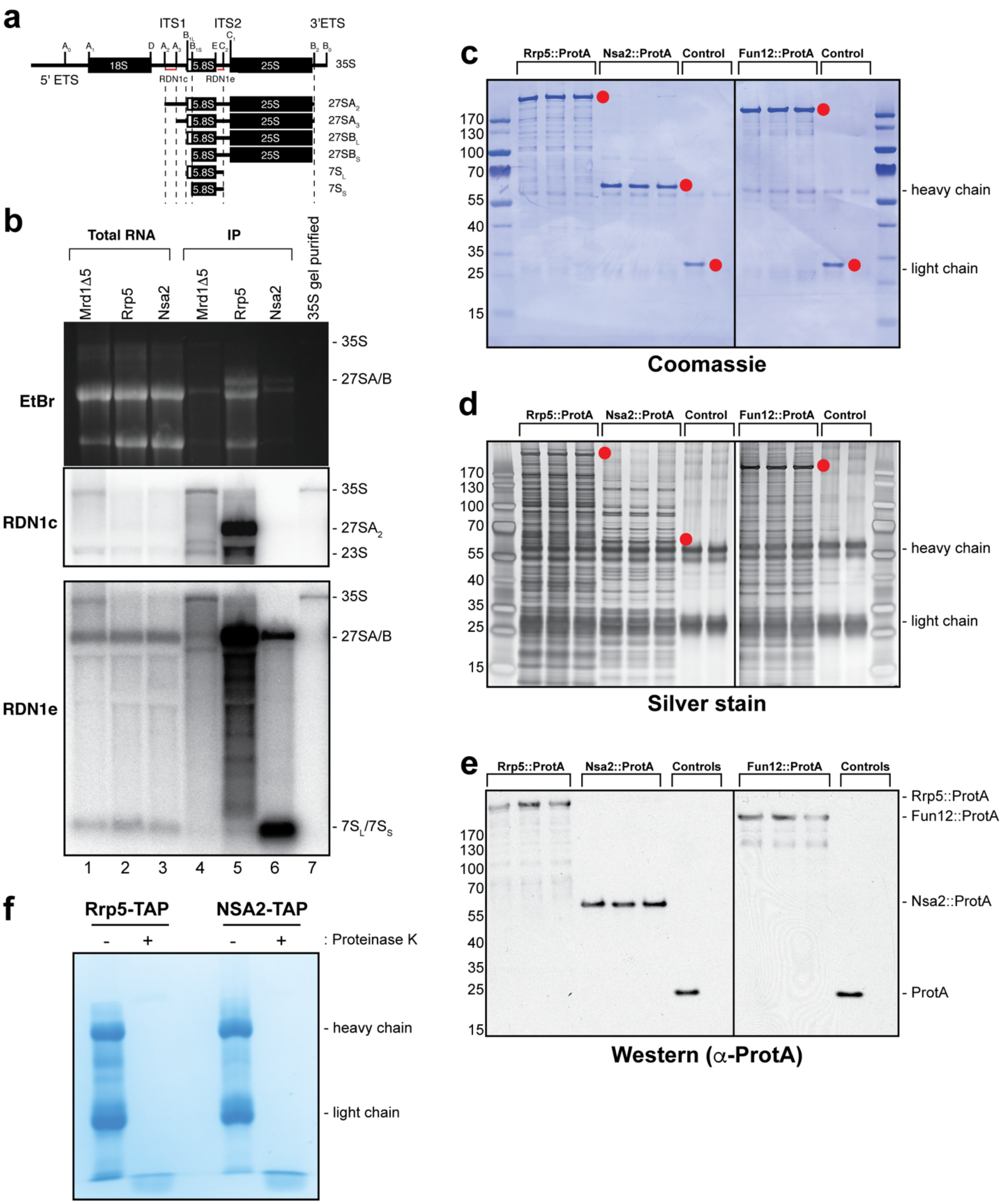
a
ITS1
ITS2
3'ETS
5' ETS
18S
5.8S
25S
35S
RDN1c
RDN1e
27SA2
27SA3
27SBL
27SBS
7SL
7SS
b
Total RNA
IP
Mrd1Δ5
Rrp5
Nsa2
Mrd1Δ5
Rrp5
Nsa2
35S gel purified
EtBr
RDN1c
RDN1e
- 35S
- 27SA/B
- 27SA2
- 23S
- 7SL/7SS
1 2 3 4 5 6 7
c
Rrp5::ProtA
Nsa2::ProtA
Control
Fun12::ProtA
Control
170
130
100
70
55
40
35
25
15
- heavy chain
- light chain
Coomassie
d
Rrp5::ProtA
Nsa2::ProtA
Control
Fun12::ProtA
Control
- heavy chain
- light chain
Silver stain
e
Rrp5::ProtA
Nsa2::ProtA
Controls
Fun12::ProtA
Controls
- Rrp5::ProtA
- Fun12::ProtA
- Nsa2::ProtA
- ProtA
Western (α-ProtA)
f
Rrp5-TAP
NSA2-TAP
- + - + : Proteinase K
- heavy chain
- light chain

Supplementary Figure 1. Purification of ribosome assembly intermediates

(**a**) Overview of pre-rRNA processing intermediates. The 35S intermediate shows the location of the pre-rRNA cleavage sites, the location of the various spacers (5'ETS, ITS1, ITS2, 3'ETS) and the location of the mature rRNA sequences (5.8S, 18S and 25S). Positions of oligonucleotide probes used for Northern blotting (RDN1c and RDN1e) are indicated with red lines. The shorter fragments indicate processing intermediates. The names of these intermediates are shown to the right of each fragment.

(**b**) Northern blot analysis of total RNA and pre-rRNA isolated from affinity purified particles. RNA was resolved on an agarose gel, visualized by Ethidium bromide staining (EtBr) and subsequently transferred to a nylon membrane and hybridized in succession with indicated probes. Various (pre-)rRNA species are indicated on the right side of each panel.

(**c**-**e**) Analysis of protein composition of isolated particles by coomassie staining (**c**), silver staining (**d**) and Western blot analysis (**e**) using antibodies that recognize the protein-A tag of the bait proteins. Complexes were purified using magnetic IgG beads and resolved by SDS-PAGE. The red dots indicate the location of the bait proteins. Shown are the results for three biological replicates of the bait proteins and two biological replicates of control samples (cells expressing only the tag or untagged cells).

(**f**). Proteinase K treatment completely degrades purified proteins. Shown is a coomassie-stained SDS-PAGE gel of particles incubated with or without proteinase K.

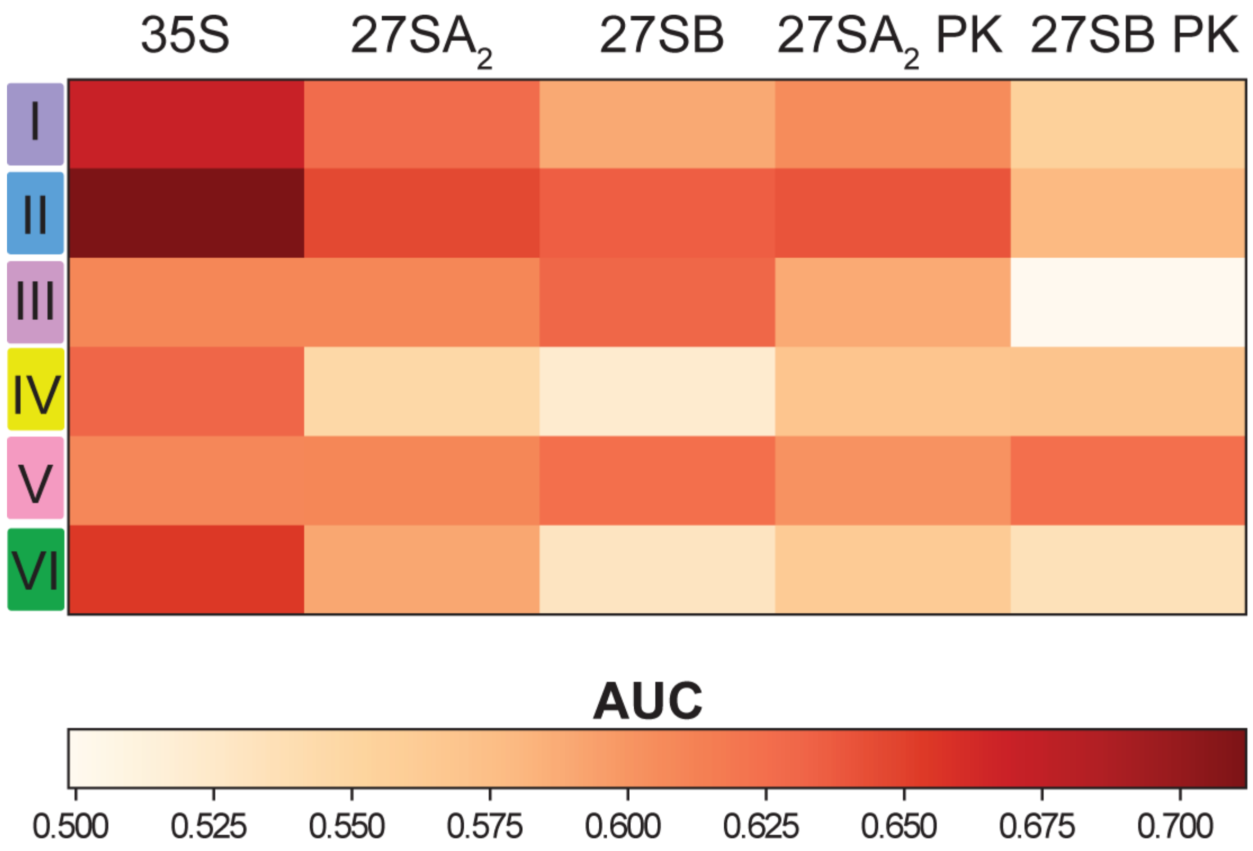

Supplementary Figure 2. Overview of ROC curve analyses for 25S domains in 1M7-probed pre-rRNA samples.

For each domain in the 25S rRNA regions (shown on the left side of the heat map), we generated receiver operator characteristic curves (ROC) where we compared the SHAPE reactivity values with the crystal structure data as binary classifier. To generate the binary classifier only nucleotides were considered that are not involved in Watson-Crick base-pairing interactions and not contacted by proteins in the crystal structure[1]. We then calculated the area under the ROC curves (AUCs) for each domain in the 25S sequence. These are displayed in the heat map. Values higher than 0.6 suggest that the data agrees reasonably well with the crystal structure data. The name of the particles is shown on top of the heat map. PK indicates particles that were deproteinized using Proteinase K.

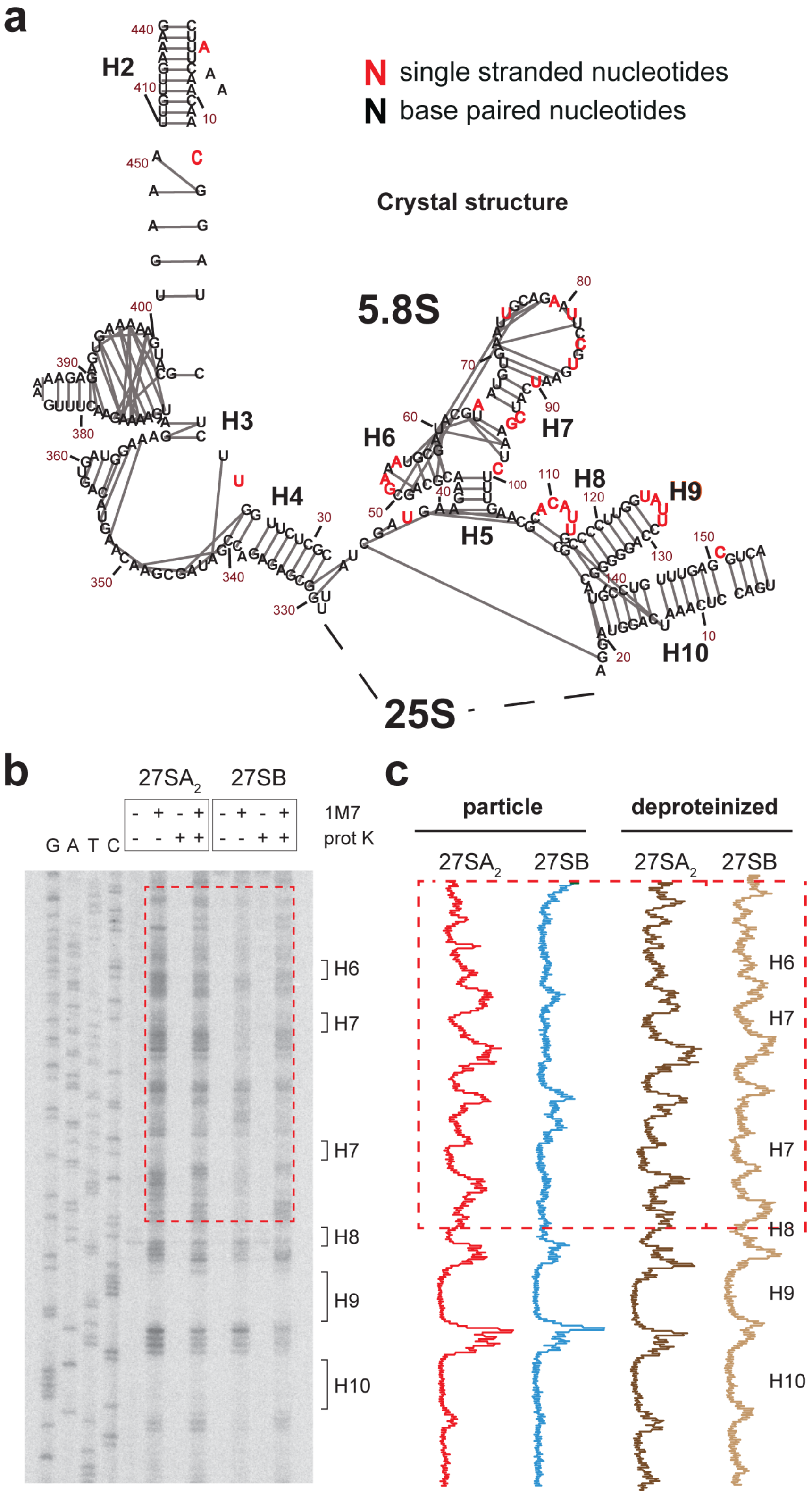
a
N single stranded nucleotides
N base paired nucleotides
Crystal structure
5.8S
25S
H2
H3
H4
H5
H6
H7
H8
H9
H10
b
27SA2
27SB
1M7
prot K
G A T C
H6
H7
H7
H8
H9
H10
c
particle
deproteinized
27SA2
27SB
27SA2
27SB
H6
H7
H7
H8
H9
H10

Supplementary Figure 3. Structure stabilization of the 5.8S region in the 27SB pre-rRNA as demonstrated by primer extension.

(**a**) Secondary structure of the 5.8S and base-pairing interactions (lines) found in the yeast 80S crystal structure[1]. Black nucleotides are base-paired, whereas the red nucleotides are single stranded in the mature 5.8S.

(**b**-**c**) Primer extension analysis (**b**) and scans of the corresponding lanes (**c**).

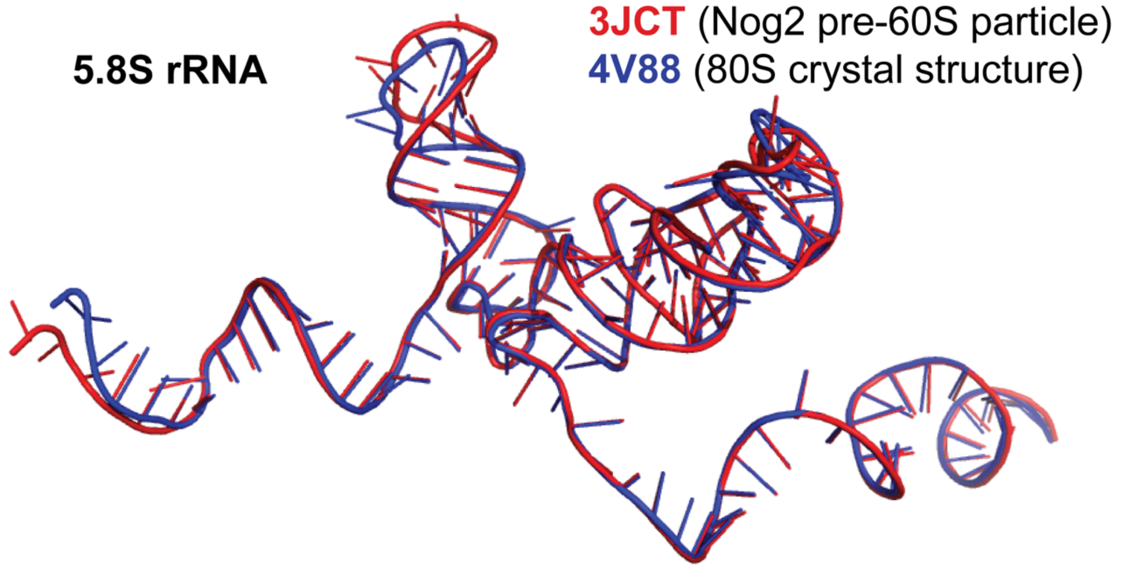

Supplementary Figure 4. The 5.8S regions in the Nog2 pre-60S particle and mature 60S have almost identical structures. Comparison of 5.8S structures in the Nog2 pre-60S particle[2] (cryo-EM; pdb file 3JCT, red structure) and the mature 60S ribosome[1] (pdb file 4V88; blue structure). The two superimposed structures appear to be almost identical.

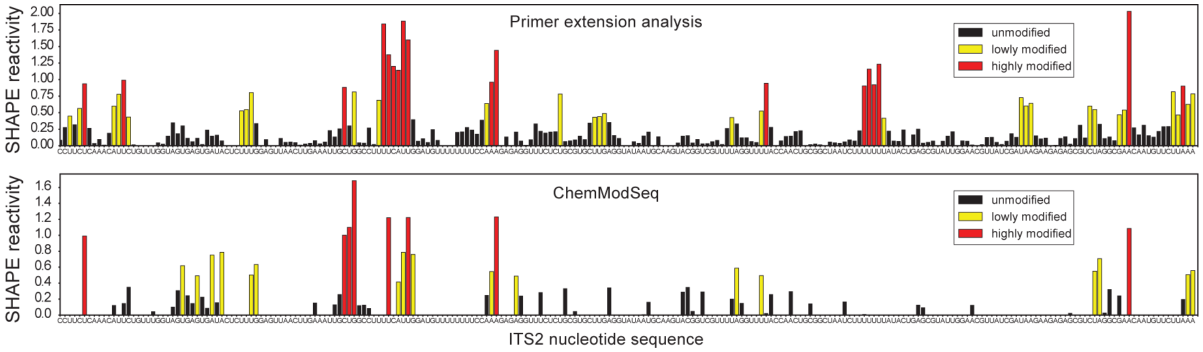

Supplementary Figure 5. Comparison of 1M7 primer extension and ChemModSeq data in the ITS2 region of $27SA_2$ particles. The barplot shows 2%-8% normalized SHAPE reactivities from primer extension and ChemModSeq data. Lowly modified nucleotides: SHAPE reactivities >0.4 and < 0.85. Highly modified nucleotides: > 0.85. Note that the last 15-20 nucleotides of ITS2 showed high variability in primer extension reactions. These nucleotides are therefore also not highlighted in Fig. 6a and 6d.

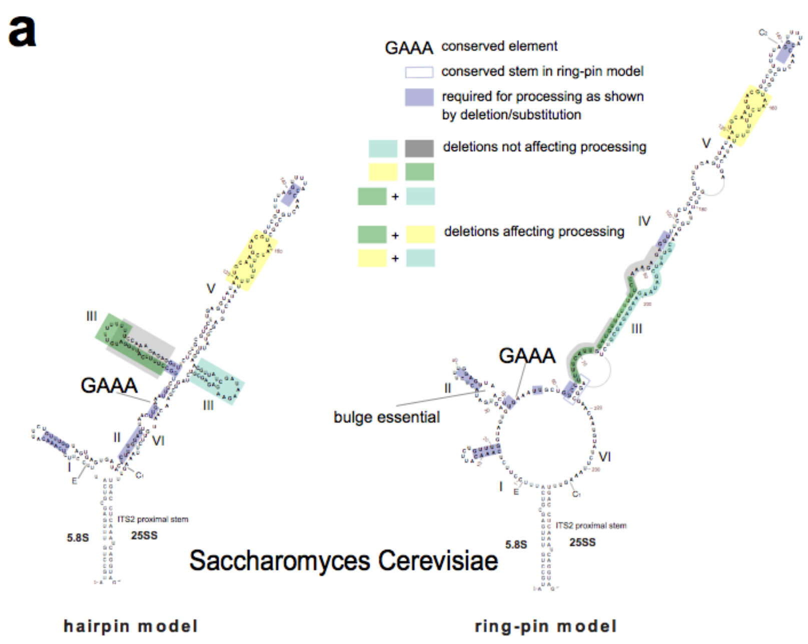

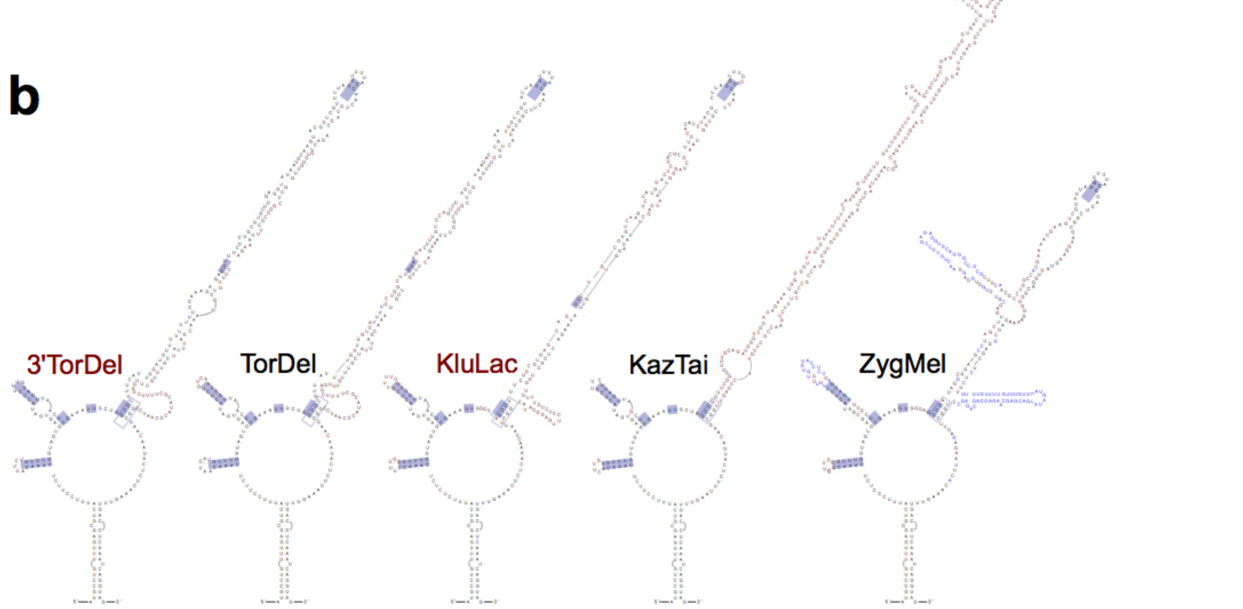

Supplementary Figure 6. Phylogenetic and functional data analysis of ITS2.

Previous ITS2 mutagenesis results are consistent with the ring-pin model as proposed by Coleman[3].

(**a**) Mutations previously tested on the hairpin model[4,5] identified regions essential for pre-rRNA processing (blue), which mostly contain highly conserved nucleotides. Deletions had addressed the role of non-conserved segments of the hairpins, which could be truncated (green, grey) or reduced in length (yellow). Combined deletions (+)

involving a shortened stem abutted by the C_2-processing site did not support ITS2 removal. Only one combined deletion, which would reduce the length of sections III and IV in the ring-pin model, supported pre-rRNA processing. A deletion in one of these strands could induce formation of a helix in the opposite strand (like in the hairpin model) and thereby be neutral. Mutagenesis supporting the ring-model demonstrated the need for the bulge in stem II[6].

(**b**) Putative ring-pin structures for various ITS2 sequences. Conserved elements emerging from phylogenetic analysis are indicated. The *Kazachstania taianensis* (KazTai) ITS2 is exceptional with respect to the length of the 'pin', encompassing regions III, IV and V in *Saccharomyces cerevisiae*. The *Zygosaccharomyces mellis* (ZygMel) ITS2 has an insertion that only allows for a ring-structure containing an extended stem-loop II. The ITS2 of *Kluyveromyces lactis* (KluLac), *Torulaspora delbrueckii* (TorDel) and a hybrid of *S. cerevisiae* and *T. delbrueckii* ITS2 (3'TorDel) had been tested in the context of a *S. cerevisiae* rDNA unit[4-6]. Only the TorDel ITS2 supported processing. The reason why the heterologous KluLac and 3'TorDel spacers did not function is not explained by an incompatible structure (i.e. change of hairpin to ring-pin model).

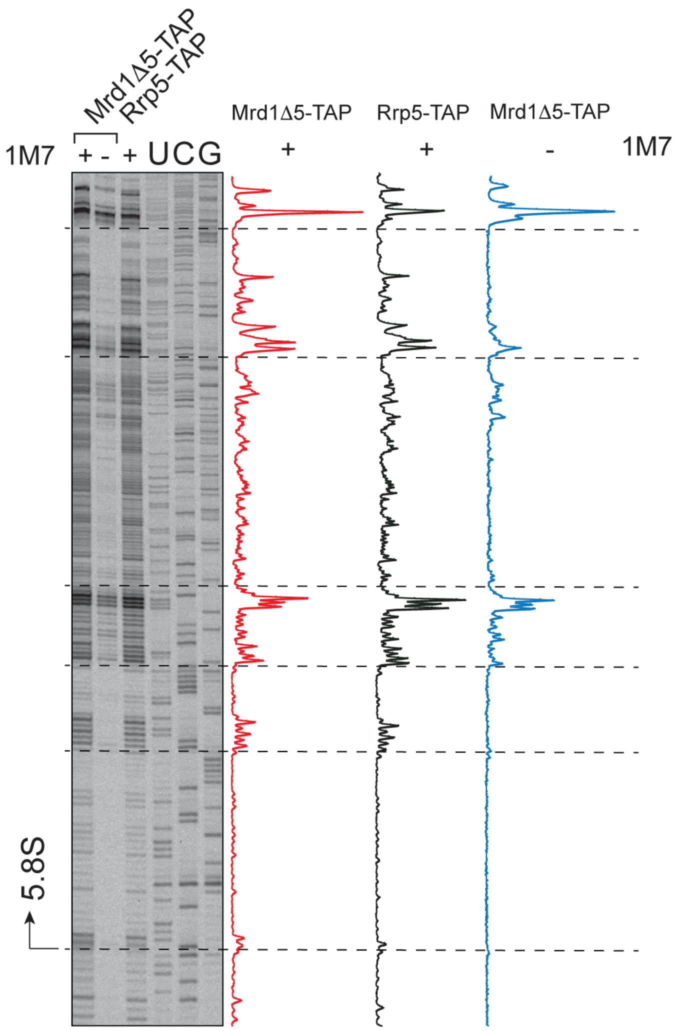

Supplementary Figure 7. Deletion of the 5th RNA binding domain (RBD) in Mrd1 does not noticeable influence folding of the 5.8S region in the 35S pre-rRNA. The primer extension results show the 1M7 probing signals in the 5.8S region of purified 35S pre-rRNA using the Mrd1 RBD5 mutant (Mrd1Δ5-TAP) or the Rrp5-TAP strain as baits. Line scans of each lane are shown on the right. UCG indicate sequencing ladders.

Supplementary Table 1.

Overview of oligonucleotides used for ChemModSeq library preparation.

Oligonucleotides for HiSeq library preparation.

RT oligo	Sequence (5’-3’)
PE_hexamer	CGTGTGCTCTTCCGATCTNNNNNN

5’ adapter	Sequence (5’-3’)
IDX1	5Phos/NCGTGATNNNNNNAGATCGGAAGAGCGTCGTGTAGGG/SpC3
IDX2	5Phos/NACATCGNNNNNNAGATCGGAAGAGCGTCGTGTAGGG/SpC3
IDX3	5Phos/NGCCTAANNNNNNAGATCGGAAGAGCGTCGTGTAGGG/SpC3
IDX4	5Phos/NTGGTCANNNNNNAGATCGGAAGAGCGTCGTGTAGGG/SpC3
IDX6	5Phos/NATTGGCNNNNNNAGATCGGAAGAGCGTCGTGTAGGG/SpC3

PCR primer	Sequence (5’-3’)
P5	AATGATACGGCGACCACCGAGATCTACACTCTTTCCCTACACGACGCTCTTCCGATCT
BC1	CAAGCAGAAGACGGCATACGAGATCGTGATGTGACTGGAGTTCAGACGTGTGCTCTTCCGATCT
BC2	CAAGCAGAAGACGGCATACGAGATACATCGGTGACTGGAGTTCAGACGTGTGCTCTTCCGATCT
BC3	CAAGCAGAAGACGGCATACGAGATGCCTAAGTGACTGGAGTTCAGACGTGTGCTCTTCCGATCT
BC4	CAAGCAGAAGACGGCATACGAGATTGGTCAGTGACTGGAGTTCAGACGTGTGCTCTTCCGATCT
BC5	CAAGCAGAAGACGGCATACGAGATCACTGTGTGACTGGAGTTCAGACGTGTGCTCTTCCGATCT
BC6	CAAGCAGAAGACGGCATACGAGATATTGGCGTGACTGGAGTTCAGACGTGTGCTCTTCCGATCT

Supplementary References

1. Ben-Shem, A. *et al.* The structure of the eukaryotic ribosome at 3.0 Å resolution. *Science* **334,** 1524–1529 (2011).
2. Wu, S. *et al.* Diverse roles of assembly factors revealed by structures of late nuclear pre-60S ribosomes. *Nature* **534,** 133–137 (2016).
3. Coleman, A. W. Nuclear rRNA transcript processing versus internal transcribed spacer secondary structure. *Trends Genet.* **31,** 157–163 (2015).
4. van der Sande, C. A. *et al.* Functional analysis of internal transcribed spacer 2 of Saccharomyces cerevisiae ribosomal DNA. *J. Mol. Biol.* **223,** 899–910 (1992).
5. van Nues, R. W. *et al.* Separate structural elements within internal transcribed spacer 1 of Saccharomyces cerevisiae precursor ribosomal RNA direct the formation of 17S and 26S rRNA. *Nucleic Acids Res.* **22,** 912–919 (1994).
6. Côté, C. A., Greer, C. L. & Peculis, B. A. Dynamic conformational model for the role of ITS2 in pre-rRNA processing in yeast. *RNA* **8,** 786–797 (2002).
