# Supplementary material for: High-throughput RNA structure probing reveals critical folding events during early 60S ribosome assembly in yeast
Source: Nat Commun. 2017 Sep 28;8:714. doi: 10.1038/s41467-017-00761-8 (PMC5620067; doi:10.1038/s41467-017-00761-8)
Supplement: Supplementary file 3 — Description of Additional Supplementary Files [file 41467_2017_761_MOESM3_ESM.pdf]

Description of Additional Supplementary Files

File Name: Supplementary Movie 1
Description: A movie derived from a pymol containing all the PKSRP sites in the 60S crystal structure of the ribosome. Each 25S domain is coloured individually. The PKSRP sites that become more constrained as the 35S is processed to $27SA_2$ (red) and as the $27SA_2$ is processed to 27SB (black) are highlighted.

File Name: Supplementary Data 1
Description: **Overview of mass-spectrometry data** See the Methods section for a detailed description of the mass-spectrometry data analyses.

File Name: Supplementary Data 2
Description: **List of 1M7 reactivities, ΔSHAPE values and r-protein binding sites for nucleotides of all 3 pre-rRNAs in the region corresponding to 27SB pre-rRNA**. The "dot Br struct" in the second column indicates the secondary structure based on the dot-bracket annotation for the corresponding nucleotides in the mature 60S ribosomal subunit[1]. Dots represent single-stranded regions, whereas brackets represent helices. The names of the helices are provided in the fourth column. A 1M7 reactivity of -999 indicates low coverage; these nucleotides were excluded from the analysis. We also provide an overview of the total coverage for each ChemModSeq library. The r-protein binding sites were obtained from the published 60S crystal structure[1] .

File Name: Supplementary Data 3
Description: **Overview of the PKSRP sites** The table contains nucleotides that show an increase or a decrease in flexibility during the conversion of 35S to $27SA_2$ and $27SA_2$ to 27SB and are bound by r-proteins in the mature 60S ribosomal subunit[1]. The domain in which the PKSRP sites were located is indicated in brackets after the nucleotide positions.

File Name: Supplementary Data 4
Description: **1M7 NMR and high-resolution mass-spectrometry data.** See methods for a detailed description of 1M7 synthesis.
