# Supplementary material for: High-throughput RNA structure probing reveals critical folding events during early 60S ribosome assembly in yeast
Source: Nat Commun. 2017 Sep 28;8:714. doi: 10.1038/s41467-017-00761-8 (PMC5620067; doi:10.1038/s41467-017-00761-8)
Supplement: Supplementary file 8 — Supplementary Data [file 41467_2017_761_MOESM8_ESM.pdf]

1-Methyl-7-Nitroistoic Anhydride (IM7)

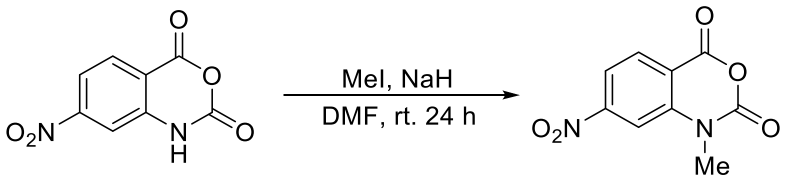

Sodium hydride (60% dispersion in mineral oil, 1.4 equiv, 5.72 mmol, 229 mg) was suspended in anhydrous DMF (13 mL) under a nitrogen atmosphere and stirred for 5 minutes. A solution of 4-nitroisatoic anhydride (1 equiv, 4.08 mmol, 850 mg) in anhydrous DMF (9 mL) was added dropwise. Methyl iodide (1.05 equiv, 4.29 mmol, 609 mg, 270 µL) was then added dropwise and the reaction stirred at room temperature for 24 h. The reaction mixture was added to partially frozen 1 M HCl (150 mL) forming a yellow precipitate. This was recovered by filtration, washed with water, then diethyl ether and dried under vacuum for 14 h to yield crude product. This was triturated from ether/pentane and dried under vacuum for a further 14 h to yield 1-methyl-7-nitroistoic anhydride (585 mg, 64%).

^{1}H NMR (500 MHz, CD_3CN) δ 8.32 – 8.29 (m, 1H, Ar ^{1}H), 8.12 – 8.05 (m, 2H, Ar ^{1}H), 3.61 (s, 3H, CH_3 ^{1}H).

^{13}C NMR (126 MHz, CD_3CN) δ 157.9 (C=O), 153.2(C=O), 147.5 (Ar ^{13}C), 143.4 (Ar ^{13}C), 131.6 (Ar ^{13}C), 117.8 (Ar ^{13}C), 116.4 (Ar ^{13}C), 110.0 (Ar ^{13}C), 32.0 (CH_3).

HRMS (ESI) obtained m/z 245.0179 (M+Na$^+$). Expected 245.0169.

^{1}H NMR

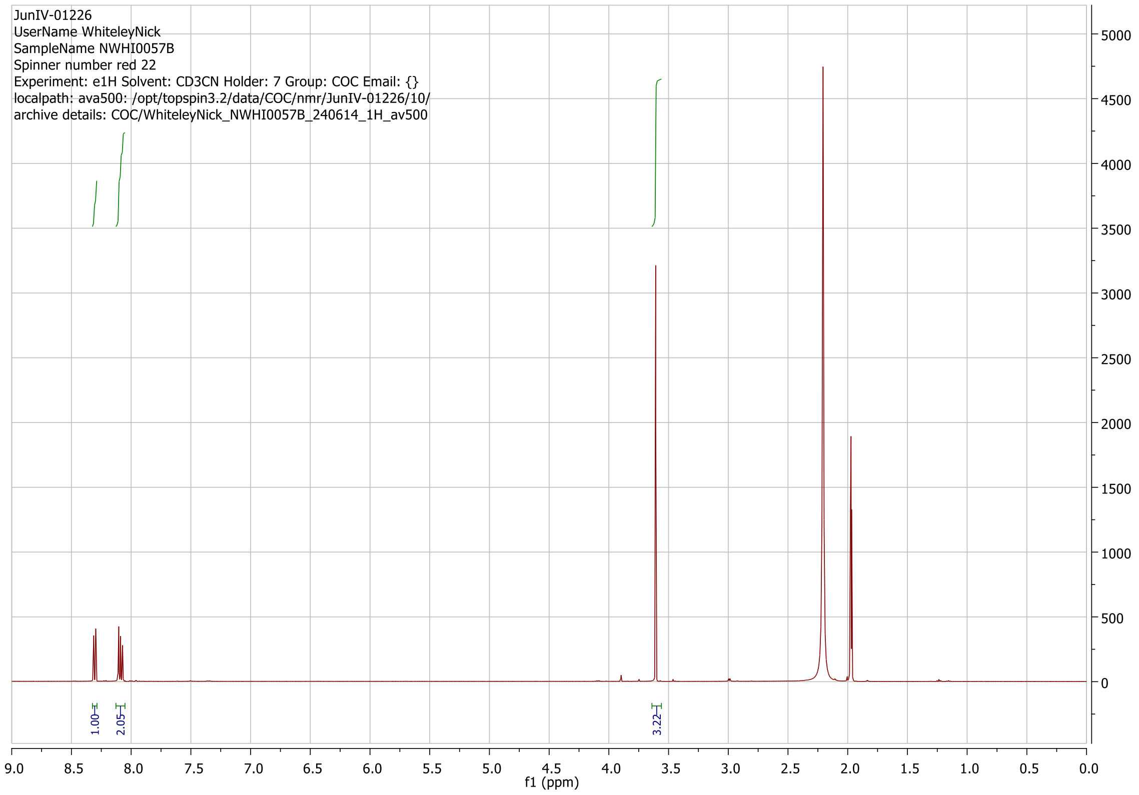

^{13}C NMR

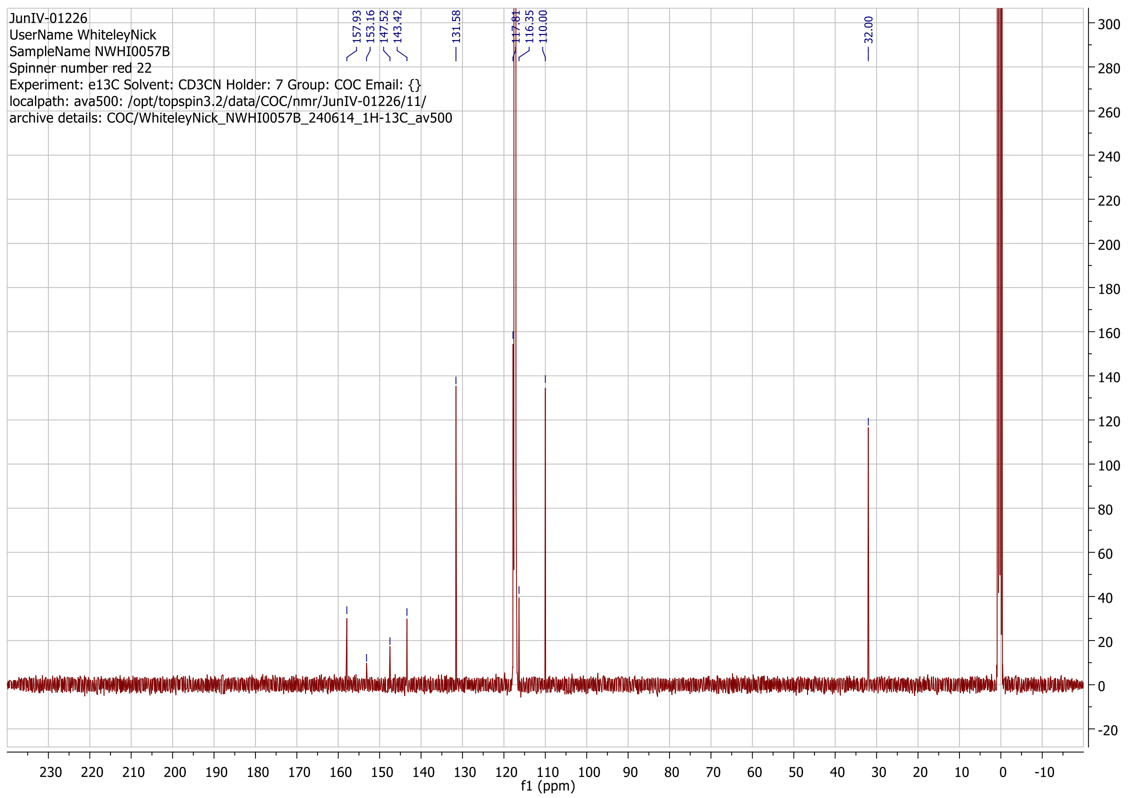
JunIV-01226
UserName WhiteleyNick
SampleName NWHI0057B
Spinner number red 22
Experiment: e13C Solvent: CD3CN Holder: 7 Group: COC Email: {}
localpath: ava500: /opt/topspin3.2/data/COC/nmr/JunIV-01226/11/
archive details: COC/WhiteleyNick_NWHI0057B_240614_1H-13C_av500
157.93
153.16
147.52
143.42
131.58
117.81
116.35
110.00
32.00
f1 (ppm)

HRMS

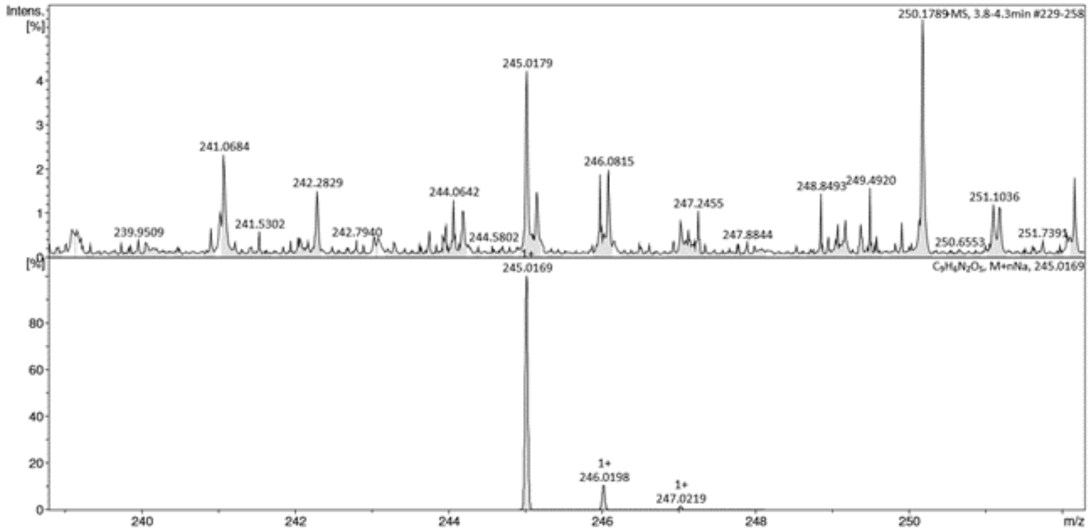
Intens. [%]
250.1789 MS, 3.8-4.3min #229-258
245.0179
241.0684
246.0815
242.2829
244.0642
247.2455
248.8493
249.4920
251.1036
239.9509
241.5302
242.7940
244.5802
247.8844
250.6553
251.7391
245.0169
C9H6N2O5, M+nNa, 245.0169
1+
246.0198
1+
247.0219
m/z
